# Risk factors for recurrent acute mastoiditis in pediatric patients: a registry-based cohort study

**DOI:** 10.1007/s00405-024-08473-8

**Published:** 2024-02-12

**Authors:** Yotam Aharon Shiner, Orit Samuel, Walid Saliba, Nili Stein, Nogah Kerem, Raanan Cohen-Kerem

**Affiliations:** 1 Department of Otolaryngology, Head and Neck Surgery, Lady Davis Carmel Medical Center, Haifa, Israel; 2 Ruth and Bruce Rappaport Faculty of Medicine, Technion-Israel Institute of Technology, Haifa, Israel; 3 Department of Community Medicine and Epidemiology, Lady Davis Carmel Medical Center, Haifa, Israel; 4 Department of Pediatrics, Bnai-Zion Medical Center, Haifa, Israel

**Keywords:** Acute mastoiditis, Recurrence, Mastoidectomy, Risk factors, Pediatric infectious disease

## Abstract

**Objective:**

To describe characteristics of pediatric patients with recurrent acute mastoiditis, and to identify risk factors for this condition.

**Study design:**

A retrospective cohort study.

**Setting:**

Data based on electronic medical records of the largest Health Maintenance Organization in Israel.

**Methods:**

Children hospitalized due to acute mastoiditis during the years 2008–2018 were identified, and their diagnosis was verified. Patients with recurrent acute mastoiditis were identified and grouped, and their characteristics were outlined and compared to those of the original group to identify risk factors for recurrence.

**Results:**

During the 11-year period, a total of 1115 cases of children hospitalized due to acute mastoiditis were identified with a weighted incidence rate of 7.8/100,000. Of this group, 57 patients were diagnosed with recurrence following a full clinical recovery. The incidence proportion of recurrent acute mastoiditis was 5.1% (57/1115), male-to-female ratio was 27:30, 73.4% were younger than 24 months, the median period from the first episode was 3.4 months (IQR 2.0;10.0), and 82.5% of the patients (*n* = 47) had a single recurrence, whereas 18.5% (*n* = 10) had two recurrences or more. Mastoidectomy and swelling over the mastoid area during the first episode were identified as the main risk factors for recurrent mastoiditis HR = 4.7 [(2.7–8.2), *p* < 0.001] and HR = 2.55 [(1.4–4.8), *p* = 0.003], respectively. Mastoidectomy was the only independent significant risk factor for recurrence in a multivariate analysis.

**Conclusions:**

Mastoidectomy and swelling over the mastoid area during the first episode of acute mastoiditis were found strongly related independent risk factor for future recurrent episodes of acute mastoiditis.

## Introduction

Acute mastoiditis (AM) is an infection of the mastoid air cells that has spread into the temporal bone. It mostly affects young children and is often preceded by acute otitis media (AOM). Many known factors contribute to AOM in children; among them are lack of proper ventilation [1] and the ability to drain pus from the middle ear cleft and thus also from the mastoid air cells that are in continuum with the middle ear cleft. The typical clinical presentation for AM is a recent episode of AOM, which is often accompanied by fever, and tenderness, erythema and swelling over the mastoid area. Some patients present with fluctuation over the mastoid area, which might indicate the presence of a subperiosteal abscess. AM is a bacterial infection that requires hospitalization, treatment with systemic antibiotics and at times with surgery secondary to extra and/or intracranial complications, therefore holds a high burden for the patients, their families, and for the health-care system.

In a recent published study by our group [2], the incidence of acute mastoiditis in the pediatric population in Israel was found to be as high as 7.8 per 100,000 children. This rate is significantly higher than previously reported in the western hemisphere, in which the incidence ranged between 0.6 and 4.8/100,000 per year [3, 4]. A few theories for this increase in incidence were suggested: Early age for attending day-care in Israel and the crowdedness in day-care facilities, population density in Israeli cities, and less antibiotics prescription for AOM due to the “Watchful waiting” approach. In that cohort, out of the 1115 patients who were diagnosed with AM, 57 patients were identified to have had two or more episodes of AM.

This study focuses on the analysis of the 57 cases who had recurrent acute mastoiditis (rAM). According to our literature search, the largest group of rAM that has been studied thus far, consisted of 32 cases, and was reported by Groth et al. [5].

The aim of our study was to better outline and assess the epidemiological and clinical features of pediatric patients with recurrent AM and to identify risk factors in order to assist in preventing future recurrent episodes which hold possible complications.

## Materials and methods

This study is based on a retrospective review of electronic medical records (EMR) of Clalit Health Services (CHS) which provides medical care for more than 4.5 million inhabitants in Israel. Data from EMR include details from the community primary and specialized clinics, pharmacies, hospitals, and medical laboratories. Diagnoses of the patients were determined using the International Clinical Diagnosis version 9 (ICD-9) specific codes.

The following data were collected from CHS medical records: demographics, vaccination status, clinical symptoms and signs at presentation, complications, imaging data, antibiotic treatment, and surgical interventions. Clinical parameters retrieved from medical files included: fever, otorrhea, otalgia, protrusion of pinna, mastoid tenderness, retro-auricular tenderness, headache, nausea/vomiting, local complications (subperiosteal abscess/other abscess), and neurological complications (meningitis, facial palsy, abducent palsy, epidural abscess, subdural abscess, and cavernous sinus thrombosis).

### Study design

Using the EMR of CHS, all pediatric patients under 18 years of age, who were hospitalized with the diagnosis of AM (ICD-9 code: 383.00) between the years 2008 and 2018 were identified. Revision of the medical record was performed to verify the diagnosis and to retrieve data. Clinical evidence required for the diagnosis for AM included AOM on admission in addition to* at least one* of the following signs: retro-auricular erythema, retro-auricular swelling, and displacement of the auricle. Supplementary data such as lab results and imaging studies were also used for adjudication. Recurrent AM were only considered as such if they were discrete. Recurrence was defined as a new episode of acute mastoiditis in the ipsilateral ear following a full clinical recovery after the initial presentation. Full clinical recovery was defined by the absence of any clinical symptoms of acute mastoiditis (e.g., presence of AOM with any clinical evidence of inflammation over the mastoid process). The minimal period between episodes to define a recurrence was 21 days. Any new presentation of acute mastoiditis within the 3 week period was considered to be a residual or a partially treated disease. Individuals were followed from the first episode of AM until the end of the study period.

### Compliance with ethical standards

The authors state nothing to disclose. There is no conflict of interests to declare.

The study was performed following approval and in accordance with the ethical standards of the Institutional Review Board of Lady Davis Carmel Medical Center) and with the 1964 declaration of Helsinki and its later amendments. The manuscript has been prepared with reference to the STROBE checklist for cohort studies (von Elm et al., Journal of clinical epidemiology 2008. https://www.equator-network.org/reporting-guidelines/strobe/).

### Statistical analysis

Continuous variables are presented as means with standard deviations (SD) or medians and inter-quartile range (IQR). Categorical variables are presented as numbers and proportions.

Differences in demographical and clinical characteristics between the recurrent and non-recurrent cases were analyzed using the Chi-square test.

Multivariate Cox proportional hazard analysis was used to identify the independent prognostic factors of recurrent AM. Hazard Ratio (HR) is presented with 95% Confidence Interval. Time to recurrence was estimated using Kaplan–Meier curves and compared between patients who had previous mastoidectomy and those who did not have mastoidectomy during their first episode of AM by log-rank test.

All statistical analyses were performed using IBM SPSS Statistics 28.0 (IBM, New York, NY). For all analyses, *p* < 0.05 (for the two-tailed tests) was considered statistically significant.

## Results

**Fig. 1:**
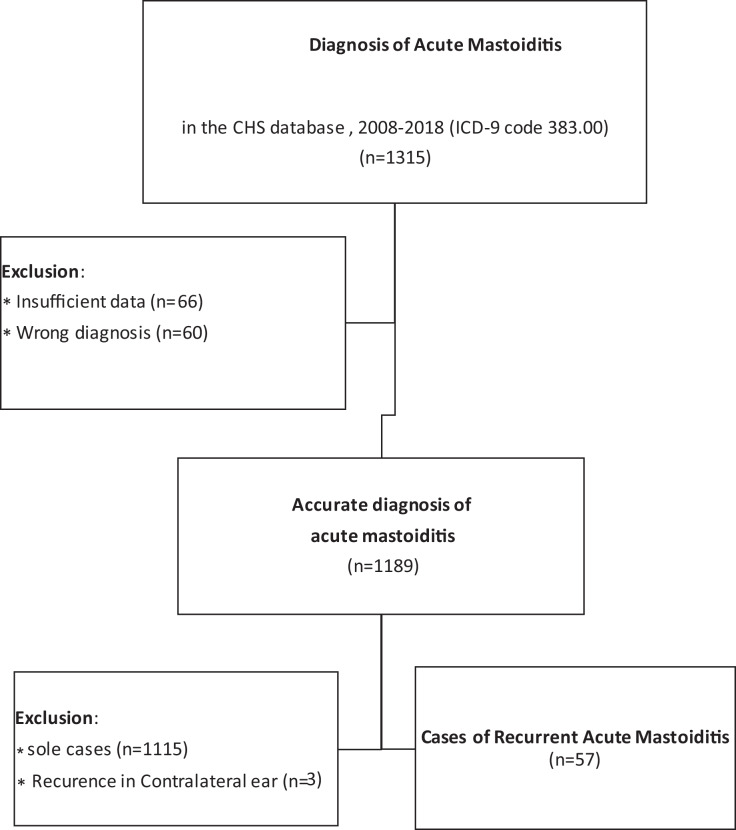
Flowchart for inclusion in the study

According to the data on the records of CHS, between the years 2008–2018, 1115 children were hospitalized with the diagnosis of acute mastoiditis. 60 patients out of this group were diagnosed with two or more discrete episodes of AM, out of which 57 patients were diagnosed with recurrence in the ipsilateral ear (Fig. 1).

The incidence of recurrent AM was 5.1% (57/1115). Median age for the first AM episode was 1.3 years [IQR (0.89; 2.5)], and male:female ratio was 27:30. The median period from the first episode to the recurrent episode was 3.4 months [IQR (2.0; 10.0)]. 82.5% of the patients (*n* = 47) had a single recurrence, whereas 18.5% (*n* = 10) had at least two secrete additional episodes. For further description of the recurrent episodes, see Table 1.

**Table 1:** Descriptive characteristics of the study population (*N* = 57)

Age (years)
Median (IQR) 1.3 (0.89–2.5)
< 2 years (73.7% *n* = 42)
> 2 years (26.3% *n* = 15)
Gender
Female (52.6% *n* = 30)
Male (47.4% *n* = 27)
Duration between episodes (months)
Median (IQR) 3.4 [2.0–10.0]
Number of recurrent episodes
1 (82.5% *n* = 47)
2 (14.0% *n* = 8)
3 (1.8% *n* = 1)
4 (1.8% *n* = 1)

**Fig. 2:**
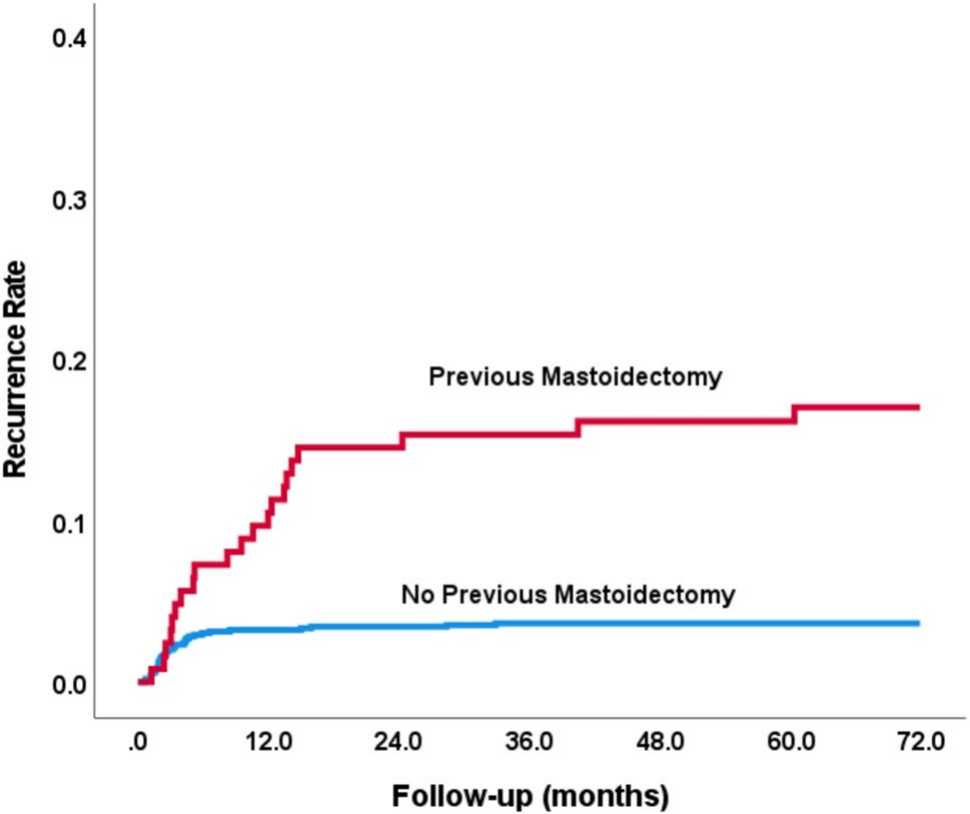
A Kaplan–Meier plot, demonstrating the differences between the periods time to recurrent mastoiditis in the patients that had undergone previous mastoidectomy (red line) and those who were treated conservatively (blue line). The difference was statistically significant (log-rank: *p* < 0.001)

Analysis for risk factors related to recurrent episodes of AM revealed that the most significant factor was undergoing mastoidectomy during the initial episode of AM: Within a year of the first AM episode, the hazard ratio for a recurrent AM episode was 3.3 among patients with AM who did not go through mastoidectomy during their first episode, in comparison to 11.3 in patients who underwent mastoidectomy. Within 6 years, this significant difference in hazard ration grew to be 17.0 in the group who underwent mastoidectomy vs. 3.6 in the group without that intervention. See Fig. 2 for the Kaplan–Meier plot of risk for recurrent episodes over years for patients with and without a history of mastoidectomy and Table 2 for the Hazard Ratio for rAM in Multivariate analysis using forward Wald model.

**Table 2:** Hazard ratio for rAM in multivariate analysis using forward Wald model

Time after first presentation with AM (months)	Previous mastoidectomy	
	Yes	No
12	11.3	3.3
24	15.3	3.4
36	15.3	3.6
48	16.1	3.6
60	16.1	3.6
72	17	3.6

Using univariate cox regression, a higher rate for recurrent episodes was detected among patients who presented with swelling over the mastoid during their initial episode [HR = 1.73, 95% CI (1.49–5.0), *p* = 0.001], patients who presented with local complication [HR = 2.2, 95% CI (1.27–3.8), *p* = 0.005], and most noticeably patients who had undergone surgical mastoidectomy [HR = 4.9, 95% CI (2.8–8.3), *p* < 0.001].

Potential other risk factors, who were found to have a *p* value of < 0.1 were: age less than 24 months [HR = 1.78, 95% CI (0.99–3.2), *p* = 0.056], high-grade fever (over 38.2 °C) at presentation of their first AM episode [HR = 1.83, 95% CI (0.92–3.62), *p* = 0.083], and neurological complication during the first episode [HR = 2.16, 95% CI (0.98–4.75), *p* = 0.057].

Mastoidectomy during the initial episode and swelling over the mastoid area during the first presentation were identified as the most significant independent risk factors for recurrent AM [HR = 4.7 (2.7–8.2), *p* < 0.001] and [HR = 2.55, 95% CI (1.4–4.8), *p* = 0.003], respectively (Table 3).

**Table 3:** Possible risk factors for recurrent AM, following univariate analysis

Risk factor	Recurrent event		*P* value
	No (*N* = 1058)	Yes (*N* = 57)	
Characteristics and symptoms at presentation			
Swelling over the mastoid process	553 (52.3%)	43 (75.4%)	< 0.001
Tenderness over the mastoid process	586 (55.5%)	24 (42.1%)	0.050
Age ≥ 24 m	414 (39.1%)	15 (26.3%)	0.053
Otorrhea	303 (28.6%)	16 (28.1%)	0.926
White blood cell count (cells per μL)	17.8 ± 6.6	18.8 ± 7.8	0.303
C-reactive protein levels (mg/L)	96.0 ± 77.6	112.5 ± 82.6	0.119
Previous treatment			
Surgical—mastoidectomy	103 (9.7%)	21 (36.8%)	< 0.001
Any surgical intervention	816 (81.3%)	48 (85.7%)	0.405
Pneumococcal vaccine	859 (94.9%)	50 (96.2%)	0.691
Complications			
Neurological complication	63 (6.0%)	7 (12.3%)	0.055
Subperiosteal abscess drainage	169 (16.0%)	13 (22.8%)	0.174

## Discussion

This study, which describes characteristics and risk factors for recurrent acute mastoiditis (rAM), includes 57 pediatric cases. It is the largest of its kind so far, describing the highest number of reported rAM cases in a single cohort. This relatively large number of cases may correlate with the higher incidence of AM described in Israel [2] when compared to the Western Hemisphere. Previous reports have suggested the possible correlation between higher rates of acute otitis media in Israel and the increased risk for rAM [6].

This study is the first yet to point out a statistically significant correlation between the surgical treatment modality for AM—mastoidectomy—during the first episode of AM, and the occurrence of further episodes of ipsilateral mastoiditis.

The etiology that accounts for a higher risk of rAM after mastoidectomy is not yet fully understood; Peterson et al. wrote about operative findings in the treatment of AM [7], and suggested that removal of the corticalis and establishment of broad communication between the middle ear and the processus mastoideus could create a potential risk for AOM that might get complicated by a further AM. This theory is not in line with other surgeons, who claim that the cortical bone is often rebuilt following mastoidectomy [8].

Since such a diagnosis might not reflect the classic case of AM that recurs and develops de novo, this hypothesis might question the accuracy of the diagnosis of rAM and nonetheless simultaneously might as well even explain the mechanism of this entity. Moreover, the fact is that in both possible scenarios, not only do the patients present with the same clinical signs and symptoms, but the treatment options also remain similar, and for this purpose, the patients will be regarded as a single group.

Another statistically significant independent risk factor for rAM that was identified in this study was the presence of swelling over the mastoid at the presentation at first. AM episode (*p* = 0.003). This might serve as an alternative explanation to the argument mentioned above regarding a new case of AOM that might mimic rAM following surgical mastoidectomy, as the swelling was predicting recurrence to begin with, and without any precedent surgical intervention in the integrity of the osseus cortex as occurs in mastoidectomy. In other words, we may speculate that the damage to the integrity of the cortical bone following mastoidectomy might cause a swelling that will mimic a rAM even when the pathology is AOM alone.

Antibiotic agents were introduced to wide use during the 40s of the last century. Their role in the treatment of AM, including decreasing rates of recurrent episodes, was described by House in 1946; he reported a rate of 20% for rAM before the introduction of sulfonamide agents, and only 5% thereafter [9]. Interestingly, although 8 decades have elapsed, since House's report with many changes in organisms causing AM and antibiotic regiments for treating it during that period—this rate remained almost similar to the recurrence rate that has been found in the present study. It is important to mention that up until that era, mastoidectomy has been the only therapeutic modality available for AM. This rate is relatively similar to other reports regarding recurrence of acute mastoiditis [5, 8].

To the best of our knowledge, the significant impact of a previous mastoidectomy as an independent risk factor for rAM is first described in the present study, which encompasses a relatively large cohort of rAM cases. Thus, this finding may require revisiting the practice of performing mastoidectomy as a treatment modality for more severe cases of AM, with minimizing it to selected cases, that did not appropriately respond to the use of antimicrobial agents.

There is a broad agreement nowadays, that uncomplicated AM may be treated with intravenous antibiotic agents (e.g., third generation Cephalosporines) and in addition to that, there is also a broad agreement between authors that performance of myringotomy (with or without insertion of a ventilation tube) should be part of the initial treatment and serves as both a diagnostic tool, to identify the pathogen for better adjustment of antimicrobial agents, and as a therapeutic procedure, to let the middle ear drain.

Nonetheless, cortical mastoidectomy is still considered to be an acceptable initial treatment for AM, especially in more complicated cases. According to Luntz et al., up to one-third of patients ultimately require surgical intervention because of the development of complications or inadequate improvement despite medical treatment [6]. This non-negligible proportion of the patients should be subject to inspection, in light of the current study findings, which suggest that this relatively common treatment modality might lead to recurrence of the disease.

Several studies have indicated that even in the presence of complications such as retro-auricular abscess, and intracranial complications such as sinus vein thrombosis, conservative management consisting of intravenous broad-spectrum antibiotic agent, myringotomy, and additional interventions (e.g., drainage or aspiration of retro-auricular abscess)—the outcomes for the patients were found to be similar [10]. The main advantage in conservative management in those cases was described as sparing the risks of potential complications caused by the surgery, such as damage to the facial nerve, CSF leak, intracranial infections, etc.

A systematic review investigating the treatment of pediatric acute mastoiditis published by Loh et al. [11] supports the findings of Mether et al. [10] that conservative therapy is as effective as mastoidectomy in producing favorable long-term outcomes, even in complicated AM cases with subperiosteal abscess. Loh et al. have also shown that conservative surgery provided similar cure rates when compared to extensive surgery. In a few proposed treatment algorithms for AM, the use of mastoidectomy was limited to selected cases with a suspected intracranial complication that were supported by imaging studies, or a prolonged failure of medical treatment [10, 12–14].

Our study sheds light on the potential long-term complication of mastoidectomy—recurrent AM—in spite of what was previously found that there are comparable short-term results for conservative treatment vs. mastoidectomy in treating AM. To minimize the risk for recurrent AM, Mierzwinski et al. have suggested an earlier and more aggressive treatment of subsequent acute otitis media with antibiotics [9].

A possible limitation to our study, which indicates a significantly higher risk for rAM after a mastoidectomy, is a possible difference in the initial clinical course of the patients that led to different treatment modalities: children who had mastoidectomy might have had a more severe illness and therefore might have had—theoretically—a greater chance for rAM to begin with.

It is important to notice that the existence of other several criteria, such as intracranial complications (e.g., sinus vein thrombosis), resistance to subperiosteal aspiration or the presence of specific microbiological pathogens, might be related to more severe disease and hence lead toward the choice of the surgical treatment modality.

In these cases, a previous mastoidectomy is more likely to be expected to begin with; however, these criteria have been considered and were found statistically non-significant factors for prediction of recurrence.

In addition, we may assume that physicians taking care of patients who were hospitalized for acute mastoiditis may be more rigorous in their follow-up, and therefore more rapidly and accurately diagnose a recurrent episode.

Lack of universal guidelines for AM treatment leads to variable approaches to treatment modalities, depending on experience of a specific physician or a specific medical center, subjective clinical impression, etc. Nevertheless, such a bias, even if it did exist in our cohort, was well taken into account in the multivariate statistical analysis, that still indicated mastoidectomy as a significant independent risk factor for recurrent episodes of AM. It is important to note that we lack information of additional hospital visits in patients that chose to be covered by a different HMO in Israel during the study period, and hence, the rate of rAM might be hypothetically even higher that appears in our outcomes.

Another possible limitation to our study is the lack of details regarding the extent of the mastoidectomy (e.g., cortical vs. radical) and the surgical technique itself. These variables might affect the recovery of the function of the tympanomastoid cavity and therefore the predisposition for rAM [15].

A hypothesis that an initial more aggressive mastoidectomy with broad attic exposure and posterior tympanotomy (as opposed to cortical mastoidectomy) might prevent recurrent cases of AM has been suggested [9], based on the rational that in a more aggressive surgical approach, the risk of an anatomic obstruction of the attic due to granulation tissue or bone is much lower, hence the risk for recurrence. However, there was not any statistical evidence that supported this hypothesis, since the sample in the study was too small [8].

Another limitation to our study is the lack of ability to differentiate rAM episodes from AOM in an operated tympanomastoid cavity. This is an integral limitation of a retrospective study that relies on a cohort with no documentation of this possibility. However, as discussed earlier, this fact does not alter the chosen treatment modality in any case.

## Conclusion

Our study outlines characteristics of pediatric patients with recurrent AM (age, gender, clinical, and lab initial presentation), and that they were/were not statistically different than pediatric patients with a single episode of AM.

The results of this study indicate that mastoidectomy, though a well-accepted first-line treatment, is an independent risk factor for recurrence of acute mastoiditis. This finding deserves careful consideration about the utility of this practice, due to the higher risk of future recurrent episodes of AM on top of the potential surgical hazards.

More research is required to indicate the impact of different surgical techniques on the risk for recurrent AM episodes.

## Data Availability

The data of this study are available from Clalit Health Services but restrictions apply to the availability of these data, which were used under the license for the current study and are not publicly available.

## Abbreviations

AM: Acute mastoiditis
rAM: Recurrent acute mastoiditis
AOM: Acute otitis media
EMR: Electronic medical record
